# Textile testing to assess the resistance to damage of long-lasting insecticidal nets for malaria control and prevention

**DOI:** 10.1186/s12936-020-03571-4

**Published:** 2021-01-19

**Authors:** Amy Wheldrake, Estelle Guillemois, Hamidreza Arouni, Vera Chetty, Stephen J. Russell

**Affiliations:** Nonwovens Innovation & Research Institute Ltd, 169 Meanwood Road, Leeds, LS7 1SR West Yorkshire UK

**Keywords:** Long-lasting insecticidal mosquito nets, Snag test, Tear test, Abrasion resistance, Wounded bursting strength

## Abstract

**Background:**

LLINs are susceptible to forming holes within a short time in use, compromising their ability to provide long-term physical protection against insect-borne vectors of disease. Mechanical damage is known to be responsible for the majority of holes, with most being the result of snagging, tearing, hole enlargement, abrasion and seam failure, which can readily occur during normal household use. To enable an assessment of the ability of LLINs to resist such damage prior to distribution, a new suite of testing methods was developed to reflect the main damage mechanisms encountered during normal use of LLINs.

**Methods:**

Four existing BS EN and ISO standards used by the textile industry were adapted to determine the ability of LLINs to resist the most common mechanisms of real-world damage experienced in the field. The new suite comprised tests for snag strength (BS 15,598:2008), bursting strength (ISO 13938-2:1999), hole enlargement resistance (BS 3423–38:1998), abrasion resistance (ISO 12947-1:1998) and new guidance around the seam construction of LLINs. Fourteen different LLINs were tested using the new suite of tests to evaluate their resistance to damage.

**Results:**

The resistance to mechanical damage of LLINs is not the same, even when the bursting strength values are comparable. Differences in performance between LLINs are directly related to the fabric design specifications, including the knitted structure and constituent yarns. The differences in performance do not primarily relate to what polymer type the LLIN is made from. LLINs made with a Marquisette knitted structure produced the highest snag strength and lowest hole enlargement values. By contrast, LLINs made with a traverse knitted structure exhibited low snag strength values when compared at the same mesh count.

**Conclusions:**

Prequalification of LLINs should consider not only insecticidal performance, but also inherent resistance to mechanical damage. This is critical to ensuring LLINs are fit for purpose prior to distribution, and are capable of remaining in good physical condition for longer. The new suite of test methods enables the performance of LLINs to be assessed and specified in advance of distribution and can be used to establish minimum performance standards. Implementation of these testing methods is therefore recommended.

## Background

LLINs are physically protective products for malaria control and prevention, safeguarding the health of millions of vulnerable people. They are intended to remain effective for 3 years, which means retaining physical integrity as well as insecticidal functionality. However, LLINs are widely known to accumulate holes within the first few years of use, undermining physical protection, compromising the safety of the product, and putting users at risk of malaria [1–6].

Appropriate quality specifications are essential for textile products to be fit for purpose [7]. It is normal practice for personal protective equipment (PPE) and other healthcare products made of textiles to be based on quality specifications that reflect the full range of technical attributes governing performance. Not least for health and safety reasons, these attributes are normally assessed in the laboratory prior to use of the product.

Whilst laboratory testing is an integral part of ensuring LLINs meet quality specifications for insecticidal efficiency, the same cannot be said for physical integrity. For years, the World Health Organization (WHO) Global Malaria Programme (GMP) implemented bursting strength testing as the only LLIN performance attribute directly related to physical integrity prior to distribution in the field. However, as a sole performance attribute related to net strength, it is well known to be a poor indicator of overall resistance to damage in the field [8]. The WHO prequalification programme is now looking at expanding testing to ensure specifications are fit for purpose. Not surprisingly, numerous prospective and retrospective studies report unsatisfactory LLIN survivorship in the field, with nets deteriorating to the extent that physical protection is lost in less than 3 years [9–13].

Following the formation of the Vector Control Group of the WHO Prequalification Team (PQT-VC), it has been recognized that reliance on bursting strength testing alone is insufficient and wider laboratory assessment of LLIN performance is needed. Any new textile test should of course reflect the real damage mechanisms that LLINs encounter in real use conditions.

The primary causes of physical deterioration in existing LLINs have recently been systematically elucidated [14]. An analysis of 525 nets retrieved from five different countries across Africa and south east Asia revealed that mechanical damage is responsible for 81.5% of the holes present in LLINs [15]. Seam failure leads to very large holes, and is mainly the result of poor manufacturing practice, whereas snagging, tearing, hole enlargement during use and abrasion are forms of mechanical damage related to the specification of the LLIN fabric and the nature of its constituent polymer and yarn components. The majority of the mechanical damage to LLINs was found to be consistent with normal household use [15] and the fact that currently available LLINs have inherently low resistance to such damage.

These findings confirmed an earlier study [14] that also highlighted the predominant role of mechanical damage in causing holes in LLINs made of multifilament polyethylene terephthalate (PET) or monofilament polyethylene (PE) yarns. Snagging is the most frequent cause of LLIN damage during use, and is a consequence of LLINs being caught on solid or pointy objects, causing one or more yarns in the net to break. Similarly, tearing causes multiple sequential yarn breakages, and abrasive wear leads to breakages due to progressive removal of material from the constituent yarns. It was also confirmed that small hole defects can enlarge over time to produce much bigger holes, depending on the textile structure of the LLIN [15].

Exposure to mechanical damage, such as snagging, tearing, abrasion and hole enlargement, can be considered inevitable, bearing in mind the design of current LLINs and the circumstances under which they are expected to be used by households following distribution. Accordingly, the degree to which LLINs accumulate damage will be affected by their inherent resistance to these same primary sources of damage.

Only a limited number of studies have attempted to address the need for new textile testing methods, specifically for assessing the performance of LLINs in terms of their resistance to damage. For determining snag and tear strength, Skovmand and Bosselmann [16] described a method to measure the force needed to pull a metal hook through the planar structure of a LLIN to generate a large hole. In a separate study, Smith [17] evaluated the testing of LLINs based on the wounded bursting test, and evaluated a potential hole enlargement test method by harnessing the lissajous motion of the Martindale abrasion tester.

Given the primary importance of snags, tears, abrasion and hole enlargement in the physical deterioration of LLINs during household use, the purpose of the present work was to identify a coherent suite of tests, based on standard textile industry laboratory methods that would be capable of assessing the resistance of LLINs to these threats.

## Methods

**Fig. 1:**
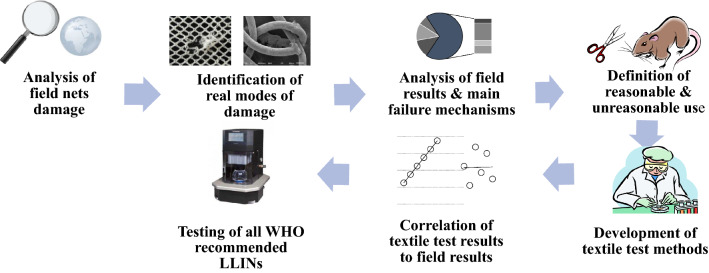
Systematic approach to identify textile test methods suitable for assessing LLIN resistance to damage

In the field of textile science, a large number of standard test methods are routinely utilized to evaluate physical properties and the performance attributes of fibres, yarns, fabrics as well as final products. Based on a careful review of available methods, combined with detailed insights of observed LLIN field damage evidence and their underlying mechanisms [14, 15], it was possible to identify appropriate test methods for LLIN assessment as part of a systematic process (Fig. 1). Test methods were selected based on existing BS EN and ISO test standards used by other sectors of the textile industry and where necessary slightly adapted to enable assessment of LLINs.

### Snag strength test

**Fig. 2:**
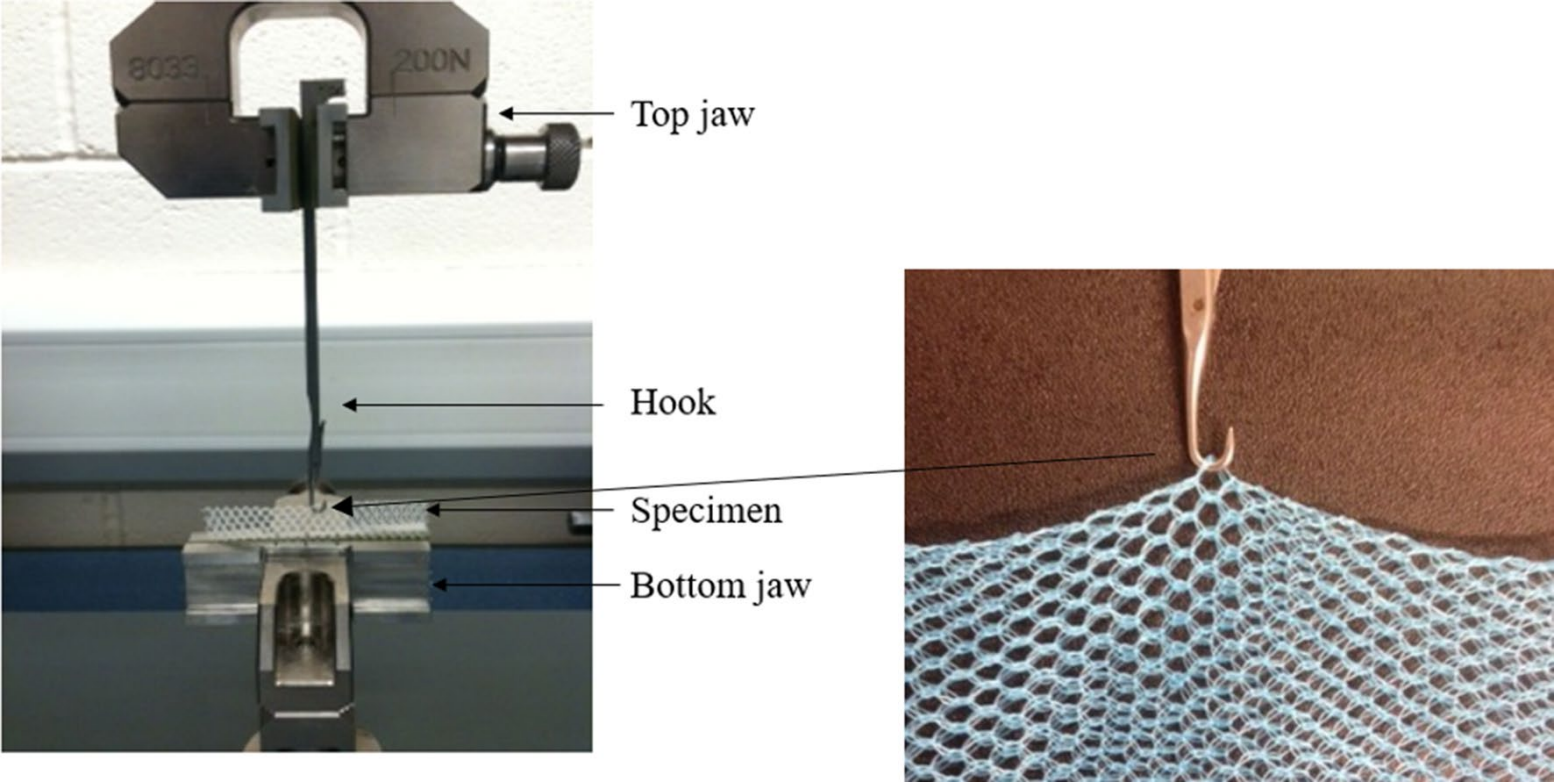
Snag strength test set-up based on BS15598:2008 (Test method for the determination of the resistance to pile loop extraction)

Snagging is the most frequently encountered form of mechanical damage in LLINs, and is caused by the mesh of the fabric catching on solid protuberances in the household, which when pulled away can lead to yarn breakage and the formation of a hole [18]. As evidenced by previous studies of field net damage [15] this can be associated with relatively small hole defects, and not necessarily large ruptures. BS 15598:2008 is a standard test method for fabrics to determine the resistance to loop pile extraction, i.e. the force is applied to the fabric perpendicular to the surface plane, similar to a plucking or pulling action. A progressive force is exerted on a single loop until it loosens and pulls along a length of successive loops at an elongation rate of 100 mm/min. This standard test can be readily adapted to measure the snag strength of LLINs as illustrated in Fig. 2. The instrumental arrangement is described in BS 15598:2008 (Fig. 2). Herein, for LLINs, it was found that a standard 3.5 gauge latch knitting needle was suitable for use as the hook. The elongation rate was increased to 100 mm/s, to be consistent with the pulling mechanism typically responsible for snagging.

**Fig. 3:**
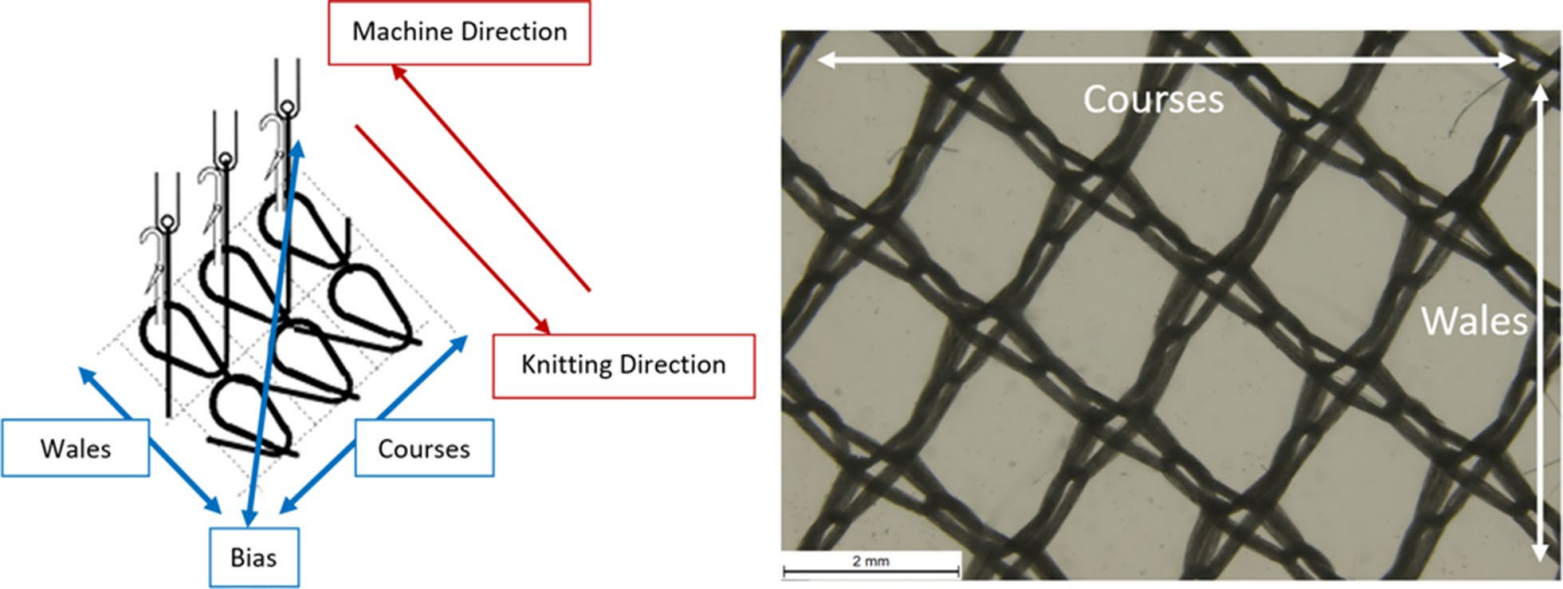
Directional properties of warp knitted structures

A tensile testing machine equipped with a stress–strain recording instrument was employed. The hook was mounted in the top jaw so its tip was 20 mm away from the bottom jaw. For testing LLINs, a fabric specimen of 100 mm × 120 mm is folded in half along its longer side and clamped into the bottom jaw so that an intersecting area of the net is positioned in the hook but is not under tension. The force needed to break the yarn in the fabric as the hook pulls the loop away from the fabric surface is recorded as the snag strength. Separate measurements (n = 15 replicates) were made in both the wale and course directions to reflect the fact that LLIN fabrics are anistotropic. Distinction of wales and courses in a warp knitted structure are shown in Fig. 3.

### Bursting strength test

Bursting strength is an existing standard method already used for determining the strength of knitted LLIN fabrics. The testing procedure and number of replicates was followed as detailed in ISO 13938-2. Although the mechanism of loading in the bursting strength test does not reflect the way in which LLINs typically deteriorate during normal use, it still provides a useful assessment of fabric strength and was therefore included in the new suite of tests. The fabric is clamped over the top of an expanding diaphragm by a clamping ring. The underside of the diaphragm is then gradually inflated with compressed air creating a dome shape, such that the overlying fabric becomes distended as it attempts to accommodate the increased dimensions. Eventually, the fabric bursts, and the bursting strength (pressure to break) and distension are recorded. The machine calibrates the pressure increase to achieve the test specimen burst within 20 s ± 5 s from the start of the test.

### Abrasion resistance test

**Fig. 4:**
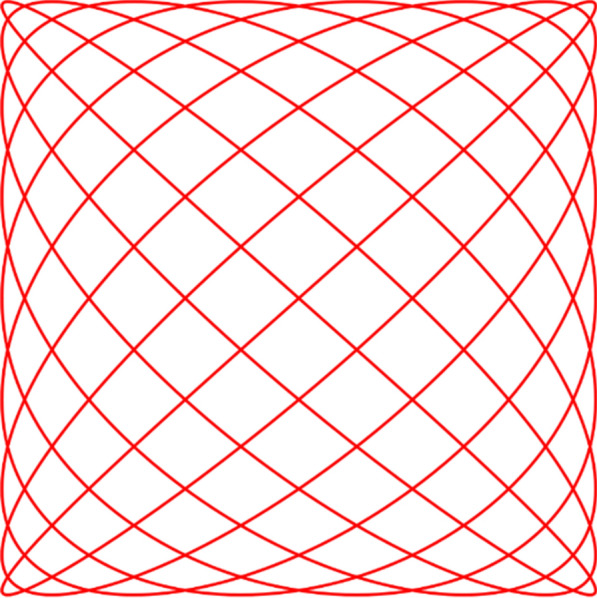
Lissajous motion across the specimen (ISO 12947-1:1998)

LLINs are regularly subjected to abrasion against other surfaces when for example, they are washed or tucked between the mattress and bedframe. The textile industry utilises a variety of standard abrasion resistance test methods but none are routinely employed for the assessment of LLINs. Use of a Martindale tester as specified in ISO 12947-1:1998 is the most common. This subjects a circular specimen of the fabric to a defined load, and rubs it against a standard abradant that traces a Lissajous figure across the surface as shown in Fig. 4.

The condition of the specimen is assessed at intervals (usually every 1000 rubs) and is stopped when an agreed end-point, such as a yarn breakage, is observed. Reaching this particular end-point using the standard Martindale abradant with a LLIN fabric can require > 10,000 rubs, resulting in a lengthy test. Replacement of the standard abradant with sandpaper is typically carried out in the testing of durable textile fabrics, such as geotextiles and protective clothing [19, 20]. Use of a sandpaper as an alternative abradant accelerates the test for LLINs and more closely mimics usage conditions, where rough wall or bedframe surfaces are encountered. Herein, a grade 7 (240-grit) sandpaper was selected as the abradant and tests were conducted in accordance with ISO 12947-1:1998 with an applied load of 9 kPa. Specimens were visually inspected at 25-rub intervals, up to 200 rubs, and thereafter at 50-rub intervals up to a total of 1000 rubs. The test end-point was defined as a yarn breakage and a resulting hole in the LLIN of at least 5 mm in diameter.

### Hole enlargement resistance test

**Fig. 5:**
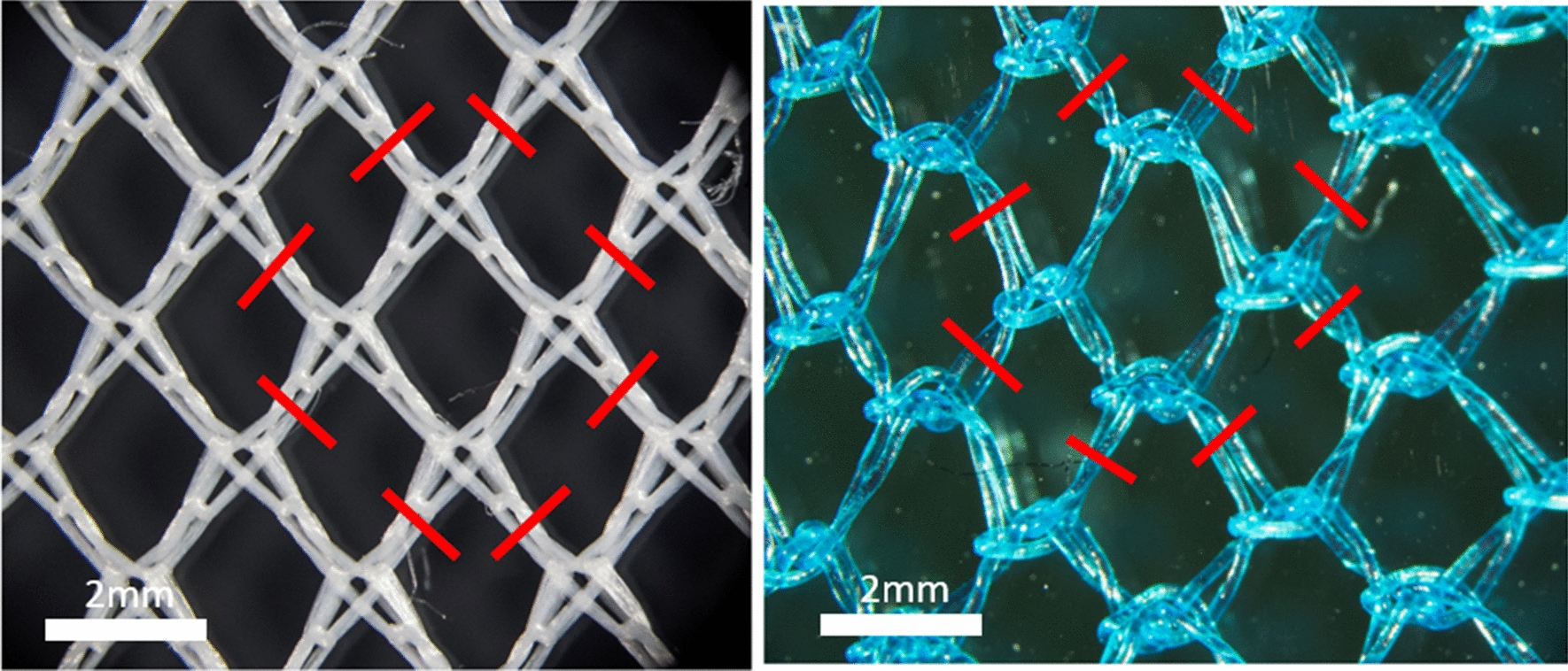
Preparation of LLIN test specimen to determine resistance to hole enlargement. Red lines represent the cutting points for removing a full mesh section prior to testing

Although holes may start small, they have potential to enlarge over time, undermining the LLINs long-term physical integrity. The hole enlargement mechanism in LLINs was carefully studied, and was effectively simulated by adaptation of the standard wounded bursting strength test method (BS 3423-38:1998). A modification was made to the way in which the test specimen is wounded prior to testing, together with cyclic loading and unloading of the specimen at a force below that needed to burst the fabric entirely. To perform this test, a unit cell of the knitted mesh in the LLIN is first removed, as indicated by the cut positions in Fig. 5.

The LLIN fabric specimen is then clamped into a standard diaphragm bursting test instrument. Herein, a Heals TruBurst unit was used with a 1.5 mm thick diaphragm, such that the wounded section is located at the centre of the 50 cm^2^ test area. A cyclical loading programme was then applied to inflate the LLIN fabric specimen to 80 kPa for three sequential cycles at a rate of 15 kPa/s, holding for 3 s in both the pressurised and relaxed states. The residual hole size at the end of the test in the LLIN fabric specimen is then determined with reference to the starting hole size (n = 15 replicates).

### Seam construction

**Fig. 6:**
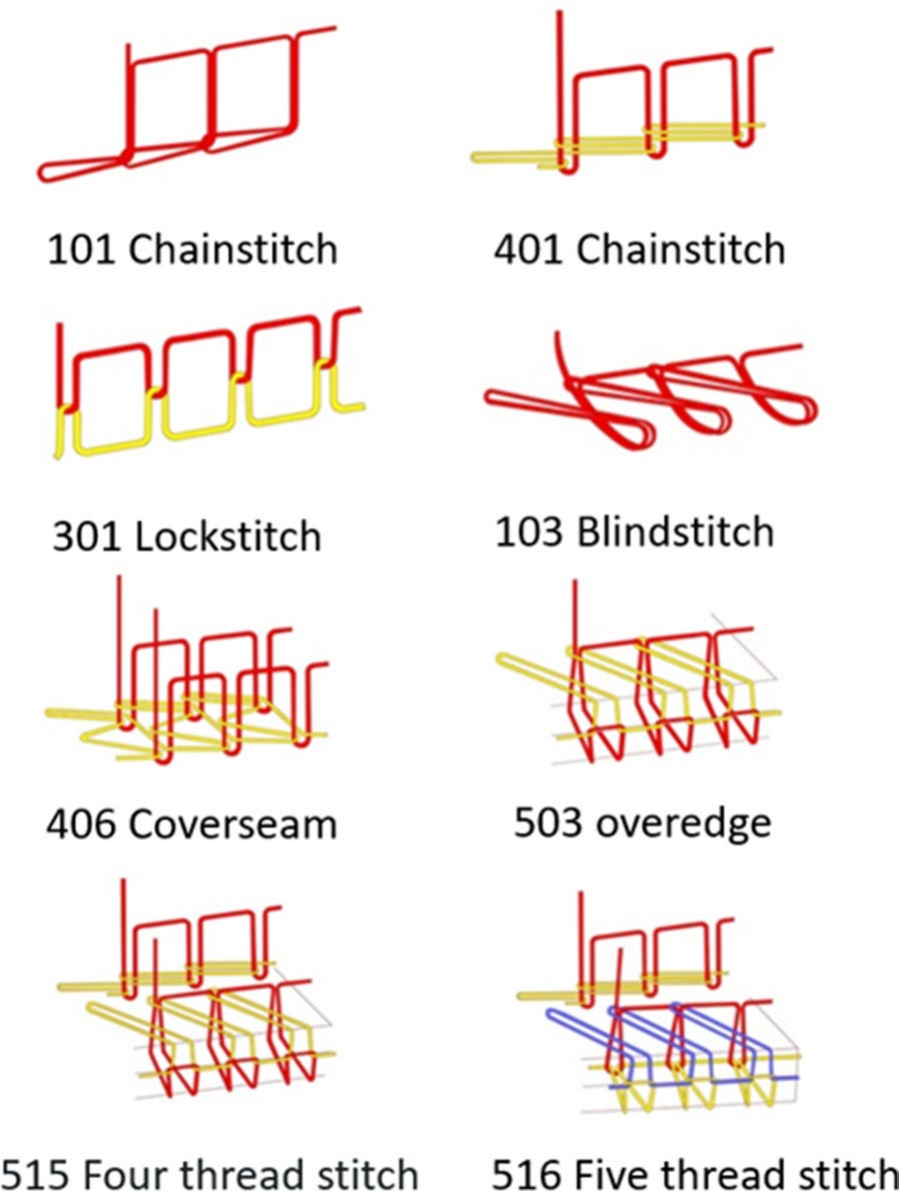
Examples of stitches used industrially [21]

The seams in LLINs are responsible for joining adjacent panels and are normally of sewn construction. The seam bursting strength for LLINs is normally measured using the standard test method (ISO 13938-2:1999) similar to the fabric bursting strength, to ensure values are similar for the LLIN fabric and seam. However, this is no guarantee of the physical integrity of the seam. Previously, it has been observed that in certain seam constructions used to manufacture LLINs, the breakage of just one sewing thread in the seam can lead to catastrophic failure as the stitch ‘runs’, causing panels to completely separate with rapid loss of physical integrity [15]. This can occur even when the bursting strength value meets the current minimum standard. Note that the use of a chainstitch (Fig. 6) combined with a low stitch density (number of stitches per unit length) makes LLINs highly susceptible to such catastrophic failure and should therefore be avoided.

### LLIN samples for testing

A total of fourteen LLINs were tested from ten different suppliers in 2013: Vestergaard Frandsen; Tianjin Yorkool International Trading Co., Ltd; Bayer industry Co., Ltd; V.K.A Polymers Pvt Ltd; Bestnet A/S; Sumitomo chemical; BASF Agro B.V. Arnhem; Clarke; Disease Control Technologies; Tana netting FZ-LLC and Shobikaa Impex Private Ltd. The various LLINs are anonymously labelled, A–N (Table 1). The warp knitted LLIN fabric structures encompassed by these products are shown in Table 2 and their details in Table 1.

**Table 1:** LLIN products tested according to developed method and interlaboratory validation carried out on LLIN products marked with an asterisk

Nets	Filament type	Knitting pattern	Areal density (gˑm^−2^)	Mesh (holes/in^2^)	Linear density (Denier)
Net A	PE monofilament	Tulle	43	56	150
Net B*	PE monofilament	Tulle	43	80	150
Net C*	PET multifilament	Traverse	41	156	100
Net D	PET multifilament	Traverse	34	156	75
Net E	PET multifilament	Traverse	42	156	100
Net F*	PP multifilament	Traverse	45	156	100
Net G	PE monofilament	Tulle	45	136	118
Net H	PE monofilament	Tulle	47	136	150
Net I	PET multifilament	Traverse	42	156	100
Net J	PET multifilament	Traverse	33	156	75
Net K	PET multifilament	Marquisette	42	169	100
Net L*	PET multifilament	Traverse	42	156	100
Net M*	PE monofilament	Tulle	50	132	150
Net N	HDPE monofilament	Tulle	50	132	150

^*^LLINs used in Interlaboratory Validation

**Table 2:** LLIN fabric structures with associated knitting pattern

		
ISO 8388:2003; 3.5.52 Tulle Warp knitted fabric with hexagonal openings produced by pillar stitches alternating with tricot stitches. Both stitches are reinforced by inlay threads (also referred to as a Raschel knit structure)	ISO 8388:2003; 3.5.50 Traverse Net Warp knitted fabric with diamond shaped openings in which each stitch in the fabric consists of only one thread (also referred to as an Atlas structure)	ISO 8388:2003; 3.5.56 Marquisette Warp knitted fabric that exhibits square openings obtained by pillar stitches that are reinforced by two sets of inlay threads lapped in opposition

### Inter-laboratory validation testing

Independently, the test methods reported herein were independently evaluated by five different textile testing laboratories located in the USA, Singapore, Portugal, UK and Germany and resulting data was collated and analysed by an independent not-for-profit academic expert [22]. The purpose was to determine if the suite of tests could be conducted by different textile testing facilities, providing repeatable results. Standard operating procedures (SOPs) for the suite of test methods were first distributed to each of the five independent test laboratories and five different LLIN branded products highlighted in Table 1 were tested according to the SOPs and specified number of replicates. The independent analysis involved a two-way ANOVA to determine whether the test methods were reproducible between laboratories.

## Results

All fourteen LLINs were assessed using the new suite of tests to determine relative performance and to develop a more detailed understanding of their inherent resistance to damage than is possible using bursting strength alone.

### Snag strength test

**Fig. 7:**
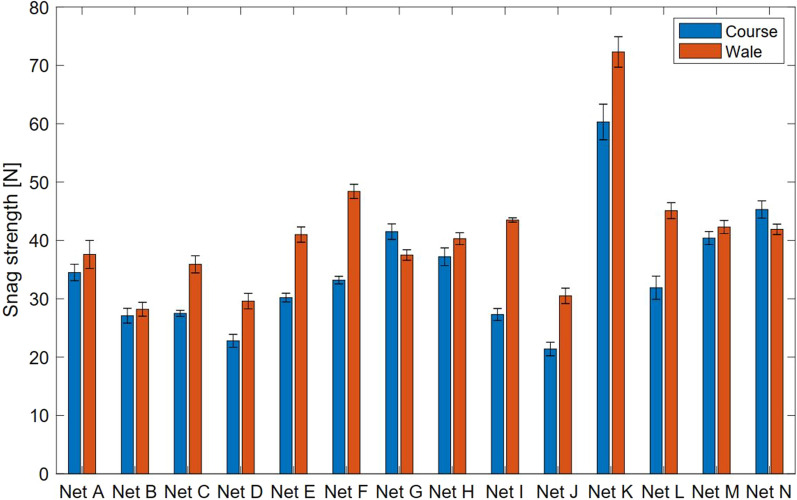
Comparison of mean snag strength values for all LLINs (A-N) based on 15 measurements per net. Error bars correspond to standard error

**Fig. 8:**
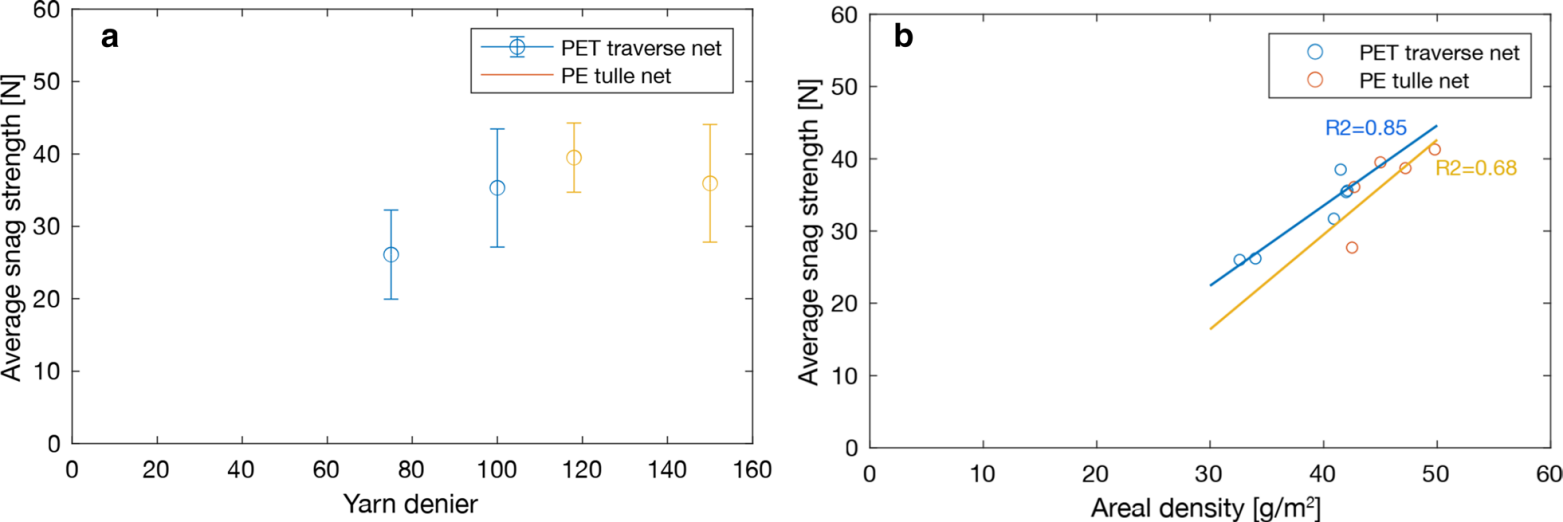
Impact of **a** yarn linear density, **b** areal density on snag strength of PET traverse nets and PE tulle nets

Figure 7 reports the snag strength test results in both the course and wale directions of the LLIN fabrics. Directional differences were anticipated because of the inherent anisotropy of knitted fabrics used to make LLINs. Higher snag strength was generally observed in the wale direction compared to the course direction (p < 0.05 for Net C–F, Net I–L), with the exception of Nets G and N, which is a function of the particular knitted fabric structures used to make the LLIN. Overall mean strength values (average of course and wale snag strength) varied from 25 to 66 N, with the best performing nets being Nets K (66.3 N), M (41.3 N) and N (43.6 N). The highest mean overall snag strength was produced by a LLIN made with a marquisette knitted fabric structure (Net K) and was significantly higher compared to the other nets (p < 0.05). Net B, Net D and Net J showed significant lower overall snag strength performance (27.7 N, 26 N and 26.2 N, respectively) compared to the other nets (p < 0.05). Nets D and J were lighter (34 g/m^2^ and 32 g/m^2^) than the rest, and were made from a relatively low yarn linear density (75 Denier), which was reflected in the snag strength performance. As shown in Fig. 8, a relation was observed between the average snag strength and yarn linear density (denier) (Fig. 8a), as well as the areal density of the fabric (Fig. 8b).

### Bursting strength test

**Fig. 9:**
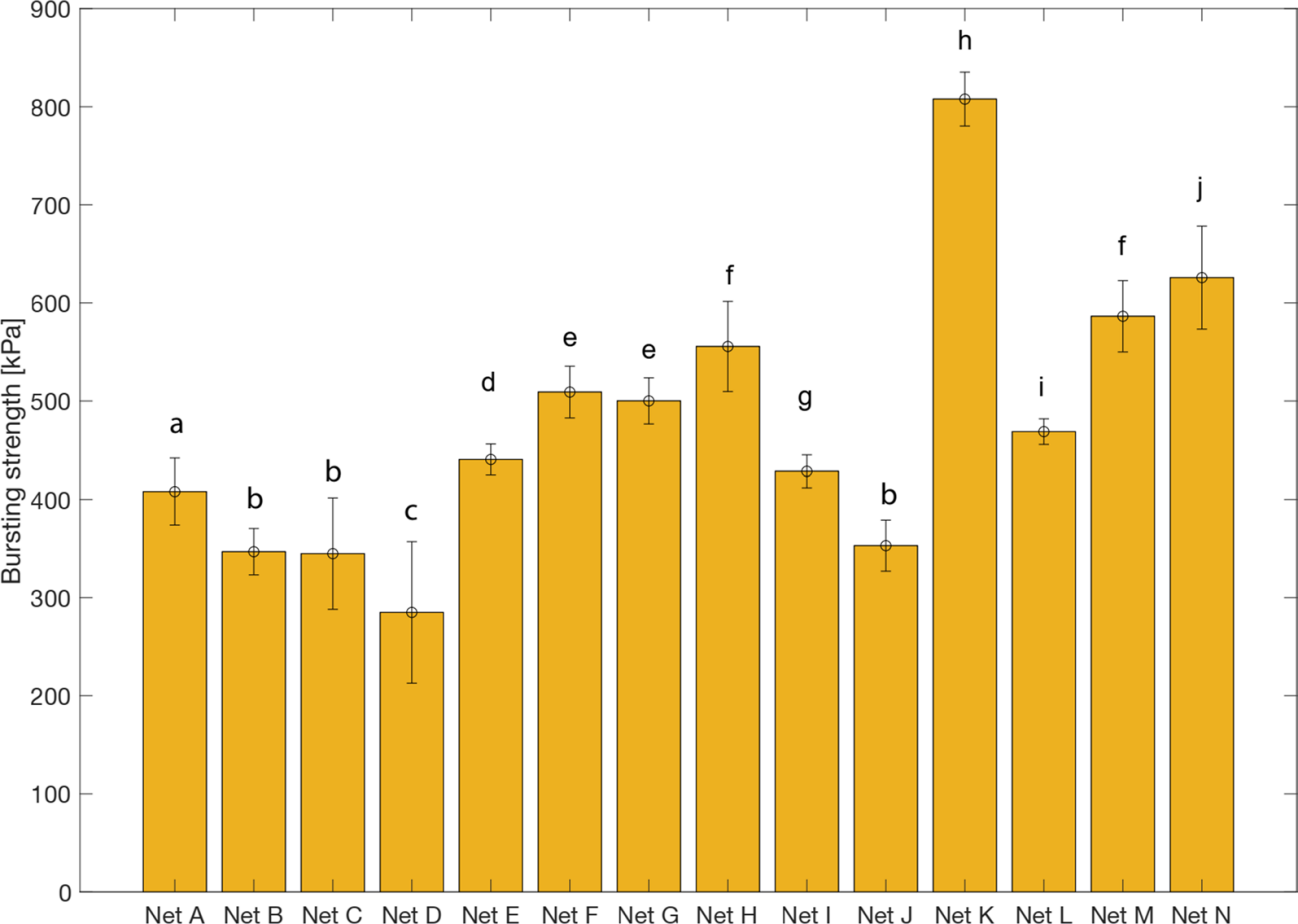
Comparison of mean bursting strength values for all LLINs (A-N) based on 15 measurements per net. Error bars correspond to standard error and values with same letter are not significantly different (p > 0.05)

The bursting strength data for the LLINs is summarized in Fig. 9. Considerable differences were observed in the performance of different branded nets. As expected, all values exceeded the currently required 250 kPa threshold value recommended by the WHO. Four net brands exhibited bursting strengths between 250 and 400 kPa, while most were in the range of 400–600 kPa. The highest bursting strength of > 800 kPa was recorded for Net K (marquisette knitted structure) and was significantly higher compared to the other nets (p < 0.05), followed by Net N with bursting strength > 600 kPa. LLINs with lower mesh count (fewer holes/inch^2^) or lower linear density (denier) exhibited the lowest bursting strength, while the highest strength was achieved by a marquisette structure. Nets with high linear density of filaments (H, M and N) exhibited high bursting strength (> 500 kPa) except for net A and B which showed poor bursting strength performance due to low mesh count (50–80 holes/inch^2^).

**Fig. 10:**
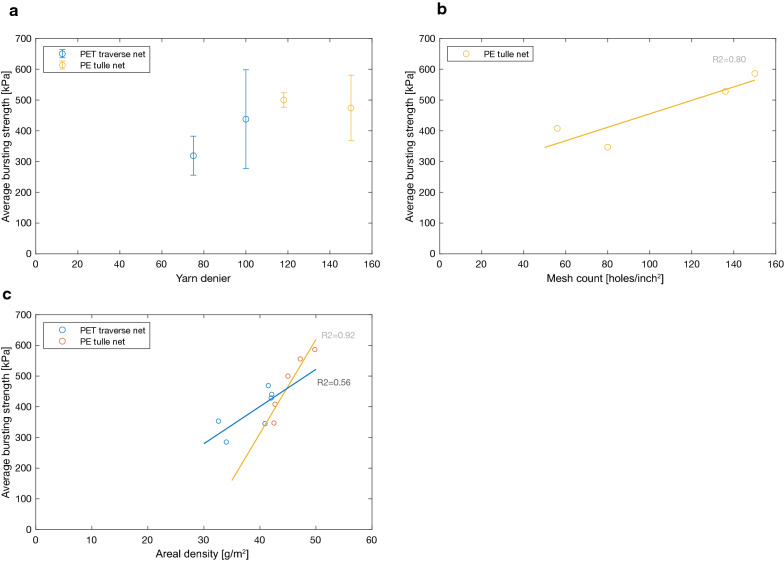
Impact of **a** yarn linear density, **b** mesh count (*excluding PET traverse net due to one mesh count) and **c** areal density on the snag strength of PET traverse nets and PE tulle nets

Bursting strength generally increased with yarn linear density (denier) (Fig. 10a) and also with increasing mesh count (Fig. 10b). The effect of increasing areal density on bursting strength however cannot be generalized, and was found to depend on the knitted fabric construction. Increasing the areal density of PE LLINs made with a tulle structure generally increased the bursting strength, but the trend was less pronounced for PET LLINs nets with a traverse knitted fabric structure (Fig. 10c).

### Hole enlargement resistance

**Fig. 11:**
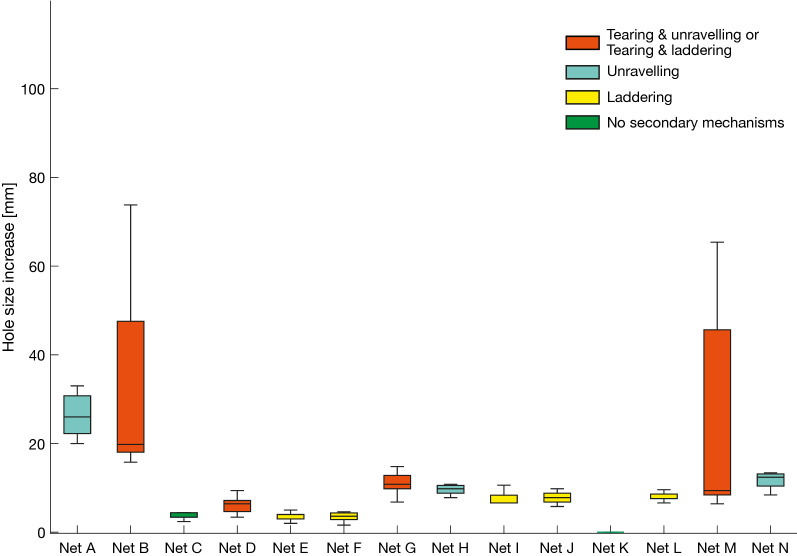
Comparison of hole size increase for all LLINs (A-N) based on 15 measurements per net. Hole enlargement data, showing degree of hole enlargement as a result of the test where, red indicates hole enlargement through two mechanisms, i.e. laddering and tearing or unravelling and tearing; blue indicates hole enlargement through unravelling; yellow indicates hole enlargement through laddering and green indicates no evidence of laddering, unravelling or tearing

The hole enlargement data in Fig. 11 characterizes the extent of laddering, unravelling and secondary tearing during bursting test. Examples of laddering, unravelling and secondary tearing are illustrated in Table 3.

**Table 3:** Secondary hole damage morphologies identified in LLINs

Type of damage	Damage morphology	Mechanism
Laddering		Enlargement of an initial hole by pulling out of successive knitted loops in the LLIN fabric structure following yarn breakage
Unravelling		Enlargement of an initial hole by unlooping of the yarns in the knitted LLIN fabric structure following initial yarn breakage
Secondary tearing		Tensile breakage of yarns within the fabric plane, in one or more directions. For example, after the LLIN is caught on a solid pointed object and then pulled in a perpendicular direction

Clearly, the largest end hole sizes were a consequence of two separate hole enlargement mechanisms operating in the same LLIN, i.e. tearing and unravelling or tearing and laddering, and some products were more prone than others. It is obvious that the degree of hole enlargement in the LLIN samples varied substantially. The most severe hole enlargement occurred when tearing (involving additional filament breakage) takes place alongside laddering or unravelling, and this was observed in four branded nets: Nets B, D, G and M. Also important is the occurrence of unravelling (with or without tearing) because it results in substantial hole enlargement. The hole size increase as a consequence of tearing varied between 7 and 33 mm with the vast majority above 20 mm while the hole size increase as a consequence of unravelling (no tearing) fell between 14 and 30 mm. As a consequence of laddering (no tearing), the hole size increase was between 3 and 8 mm, while hole size increase as a consequence of unravelling was between 14 and 30 mm. Nets C and K exhibited no laddering, unravelling or tearing during testing and their initial hole size only increased as a consequence of the fabric deformation due to the loading pressure. The hole size increase in these nets was only 3.7 mm and 0 mm, respectively, and the hole size increase of net K was significantly lower compared to the other nets (p < 0.05).

### Abrasion resistance

**Fig. 12:**
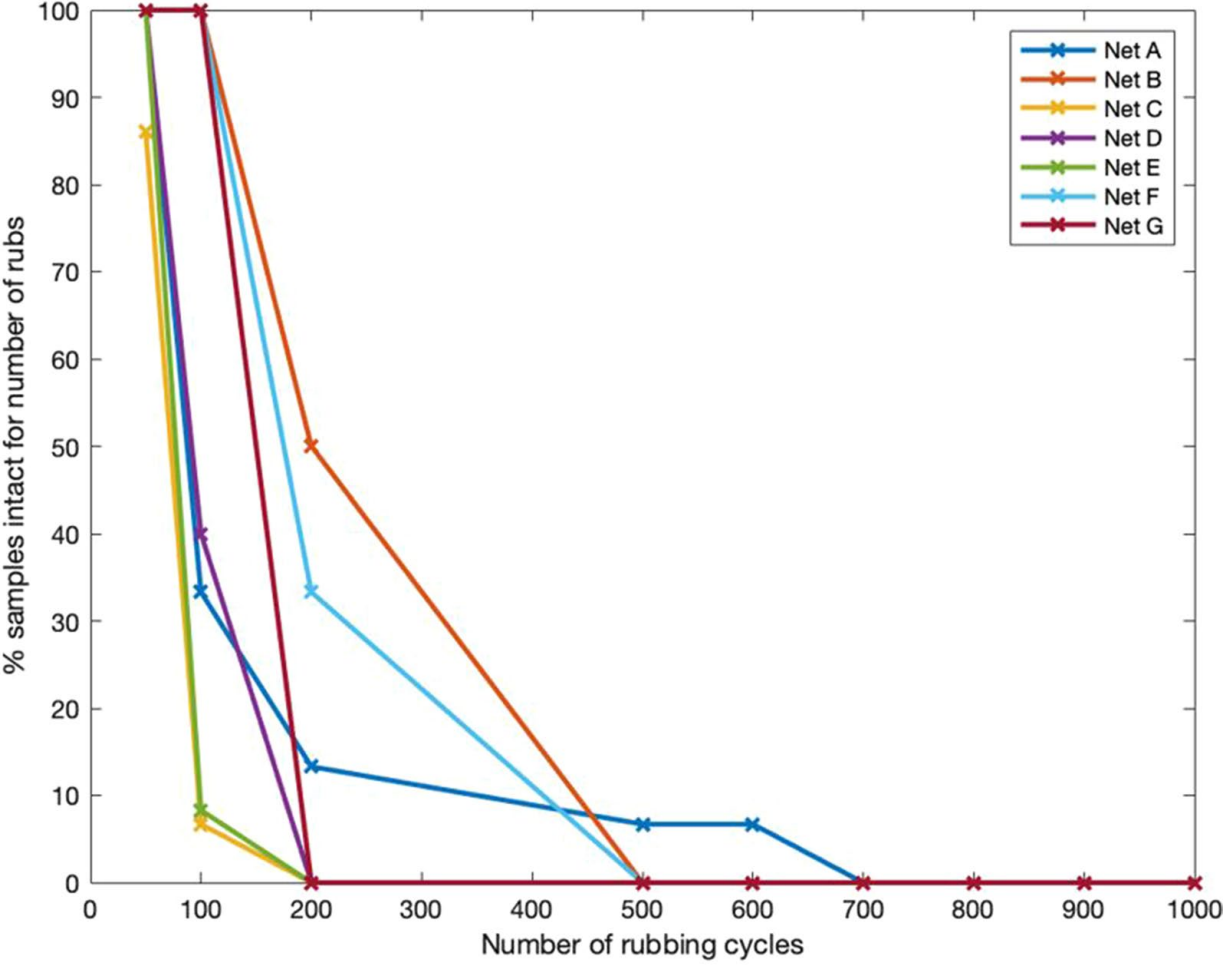
Rate of net attrition during abrasion testing (Nets A-G) at 50 to 1000 rubbing cycles

**Fig. 13:**
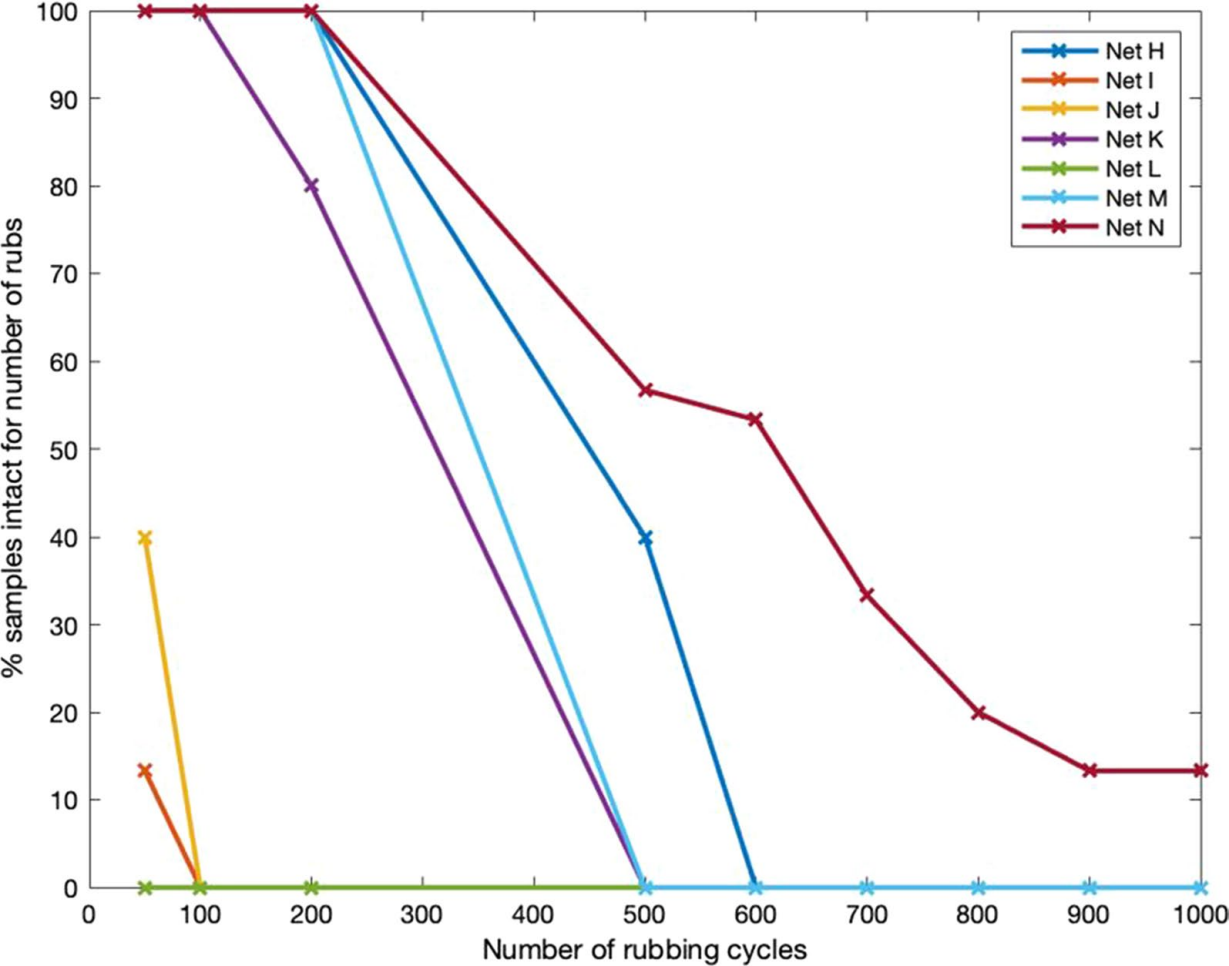
Rate of net attrition during abrasion testing (Nets H-N) at 50 to 1000 rubbing cycles

Figures 12 and 13 illustrate differences in the rate at which LLINs samples reached the test end point due to the creation of a hole of at least 5 mm in diameter. Large differences in abrasion resistance between LLINs were noted, with Net L performing particularly poorly, withstanding less than 50 rub cycles. The majority of LLINs failed at 200 rubs, with only four nets achieving more than 200 rubs (H, K, M and N). Two brands (H and M) had 5 out of 15 samples intact at 400 rubs, while net N exhibited the best abrasion resistance with 50% of specimens intact at 500 rubs and a single specimen remaining intact at 1000 rubs.

In general the LLINs made of monofilament tulle structures exhibited higher abrasion resistance than multifilament yarn traverse structures (Table 4), with the best performance associated with the PE monofilament structure (net N).

**Table 4:** Abrasion resistance of different net structure

Net structure	Percentage of samples intact for number of rubs								
	50	100	200	500	600	700	800	900	1000
Multifilament traverse	67.41	31.88	14.16	0	0	0	0	0	0
Monofilament tulle	100	88.88	60.55	17.23	10	5.55	3.33	2.22	2.22

**Fig. 14:**
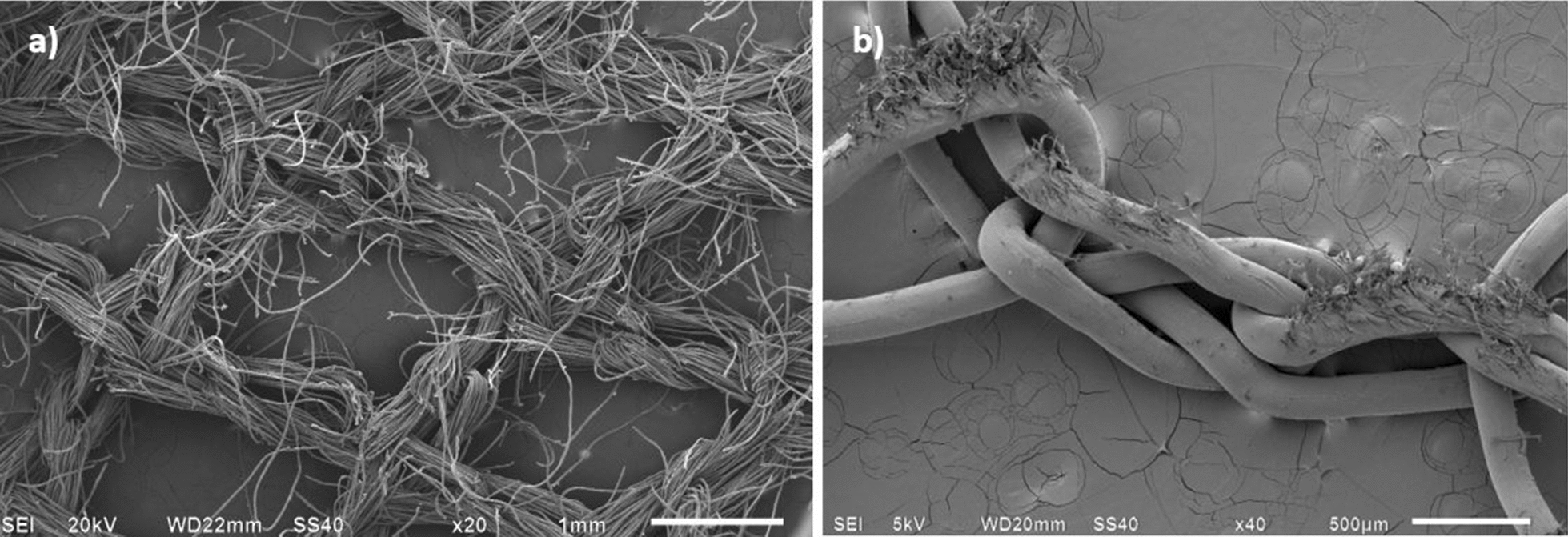
Example of **a** multifilament PET traverse net and **b** monofilament PE tulle net after abrasion

Multifilament yarns comprise bundles of much finer individual filaments within their cross-section, which permit greater inter-filament slippage during abrasion, eventually leading to protruding broken ends as is evident in the SEM image in Fig. 14a (multifilament PET traverse) as compared to monofilament in Fig. 14b (monofilament PE tulle). The relatively high mesh count in the PE tulle structure was found to have a positive impact on the LLIN’s abrasion resistance.

### Interlaboratory testing

Interlab variation testing was carried out by a separate not-for-profit academic group and the results are separately reported [22]. In the associated statistical analysis it was recognized that there will be differences in the bed net samples due to the variability in the mechanical properties of the nets, operator variation during the laboratory testing and just random variation (residual). These were separated into two factors, variability between nets and variability between laboratories. The validation of the test methods was determined by measuring the source of variability between the laboratories. The analysis reported variation below ≤ 3% as being indicative of very good reproducibility in the data between the laboratories and values between 3 and 6% were regarded as acceptable [22]. The laboratories data showed successful cross-validation of the test methods. A large proportion of the identified variations in the results was due to the type of net whilst a very much smaller proportion was due to the variation in the testing at the individual laboratories (Table 5). The interlaboratory variation for all the LLIN test methods were all consistently below 6% as indicated in Table 5, which is within the statistically acceptable limit [22].

**Table 5:** Interlab variation across the four new test methods

Source of variability	Bursting strength (%)	Snag strength test		Hole enlargement test (%)	Abrasion resistance test (%)
		Force at break (wale) (%)	Force at break (course) (%)		
Interlab	2	0	1	5	6
Net	90	81	74	79	70
Residual	8	19	26	16	24

The snag and bursting strength tests exhibited little variation across labs, 0%, 1% and 2%, in respect of the force values, with hole enlargement and abrasion resistance showing larger variation of 5% and 6%, respectively (Table 5) [22]. The higher variation is an inherent feature of tests where results are subjectively assessed by laboratory staff and hence the characterization of the output criteria subjected to interpretation by the persons who carry out the testing. The 5% and 6% variation were considered satisfactory given the partly subjective assessment involved in hole enlargement and abrasion testing [22].

### Comparison with field data

Data from each of the individual test methods were combined into a simple composite value and compared with the actual physical integrity of LLINs of the same brand observed in the field using the adjusted Proportional Hole Index (PHI) data from the study by Wheldrake et al*.* [15]. The PHI for each LLIN was calculated following WHO guidelines as summarized in Table 6 and was adjusted to exclude damage attributed to unreasonable use of the LLIN, i.e. holes attributed to animal, thermal or cut damage.

**Table 6:** WHO hole size guidelines and hole index used to assess physical integrity of LLINs

WHO 2013 guidelines	Size banding	Hole diameter	Hole radius		Area of hole	Hole Index^a^
	cm	d; cm	r = d/2; cm	r^2^; cm^2^	cm^2^	
Size 1 Smaller than a thumb	0.5–2	1.25	0.625	0.3906	1.23	1
Size 2 Larger than a thumb but smaller than a fist	2.5–10	6	3	9	28.28	23
Size 3 Larger than a fist but smaller than a head	11–25	17.5	8.75	76.5625	240.56	196
Size 4 Larger than a head	≥ 26	30^b^	15	225	706.95	576

A -area of the hole pr^2^; p = 3.142; ^a^ Area divided by 1.23; ^b^ Assumed diameter

Absolute values measured in the laboratory were summed to form a simple composite value except for the hole enlargement resistance where a scoring system was applied to calculate the absolute value (Table 7). The scoring system takes into account the end hole size and the presence of secondary damage. The larger the hole, the lower the score, while secondary damage, such as unravelling and tearing resulted in low scores due to the susceptibility to hole enlargement compared to laddering.

**Table 7:** Hole enlargement scores reflecting the size of the end hole size and type of secondary damage

Damage type	End hole size		
	< 5 mm	6–20 mm	21 > mm
None	100	80	40
Laddering	80	64	32
Unravelling	50	40	20
Tearing combined with laddering or unravelling	40	32	16

**Fig. 15:**
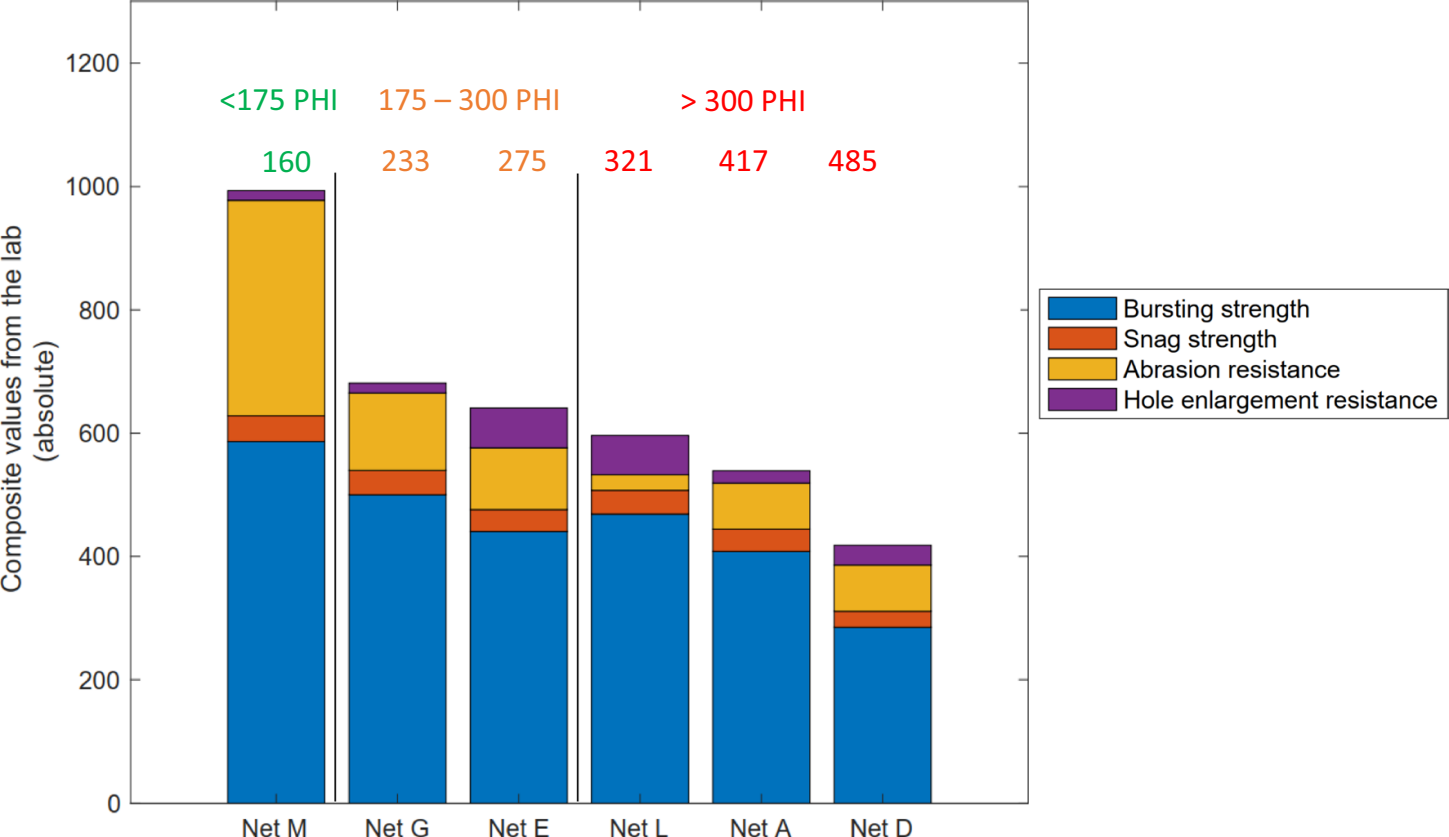
Composite value of resistance to damage (lab) vs. PHI (field) for LLINs

As illustrated in Fig. 15, comparison of the composite values obtained from the laboratory and the corresponding PHI values are in reasonable agreement. The higher the composite value, the lower the PHI, such that the performance of the LLIN in textile testing is indicative of the overall susceptibility of the LLINs to damage in the field.

## Discussion

LLINs need to remain in good physical condition for many years if they are to serve their intended purpose. As with all other textile products that protect people from harm, assessing the full range of performance attributes that directly govern their fitness for purpose is important. Generally, in the textile industry, this means rigorous laboratory testing prior to use, together with minimum performance specifications. In relation to physical integrity, it is known that LLINs are routinely exposed to various forces that cause yarns to break and holes to form and so understanding the nature of these forces, how they are resisted by LLINs, was fundamental to the development of the new suite of textile tests.

Snagging is the most common mechanism of damage in LLINs and is caused when the mesh catches on a solid protuberance or objects, and the user attempts to separate the two surfaces [15]. Skovmand and Bosselmann proposed a test method designed to assess snag strength using a tensile tester and a hook, which when pulled through the specimen, enables a very large rupture in the LLIN fabric to form, akin to a tear [16]. However, field damage evidence [14, 15] reveals that the majority of snags produce small holes (at least initially), which can be due to the force being applied perpendicular to the LLIN surface after the yarns are caught on a protruberance rather than in-plane. Indeed, the small ‘pulls’ or loops that are often seen projecting from the surface of LLINs, are further evidence of snagging in this way. Accordingly, the force that the LLIN has to resist during snagging can be highly concentrated on just a small number of yarns. Lightweight knitted fabrics such as LLINs are highly susceptible to snagging because of their open mesh structure and the relatively small diameter of the constituent yarns and filaments.

The present work revealed large differences in the snag strength of different LLIN products, that are attributable to basic differences in yarn and knitted fabric structure. LLINs with a marquisette knitting pattern exhibited the highest snag strength due to double thread reinforcement in the mesh, while LLINs with a low filament linear density (denier) and low stitch length were the weakest. Stitch density reflects the dimensional stability of the knitted structure and is related to the length of filament in a knitted loop (mesh count), filament diameter, stitch structure and wale spacing. The greater the stitch density, the greater will be the cohesion (frictional resistance) between filaments, which will improve the snag resistance [23].

Bursting strength is the standard textile test method used at present for evaluating net physical robustness. It provides, therefore, a reliable indication of the mechanical stability of nets due to tearing, i.e. where sequential breakage of yarns take place combined with load sharing during rupture of the LLIN. However, this does not reflect all of the main damage mechanisms occurring in the field. Bursting strength is a function of yarn denier, type and areal density. Skovmand et al*.* [16] have highlighted the effect of areal density and filament diameter on bursting strength. The present work confirmed that LLINs made with a low linear density yarn (75 Denier) and/or a low knitted mesh count lead to low bursting strengths and these findings are in general accordance with the observations of Skovmand et al*.* and Yesmin et al*.* [16, 24]. In practice, the areal density of the fabric is influenced by the filament diameter and mesh count, so the parameters are not independent, and need to be balanced based on performance needs and economics.

Hole enlargement in LLINs is an important mechanism by which small holes can quickly become very large. It highlights the need to resist hole formation even if the initial defect does not immediately compromise physical protection. Measuring propensity for hole enlargement following a small cut in a LLIN has been attempted based on wounded bursting testing [25] to a pre-wounded sample, as well as cyclic loading (reported herein). The benefit of cyclic loading is that the LLIN sample is not taken to the full rupture point, in contrast to the wounded bursting test, and results are not affected by an additional wear mechanism, i.e. flat abrasion, as the hole is enlarged. As is evident in the present work, resistance to hole enlargement in LLINs is strongly influenced by the choice of knitting pattern, but can also be negatively affected by a large stitch length, due to the ease of yarn displacement under applied tension. The marquisette structure exhibits a remarkable resistance to hole enlargement due to the stability of the knitted structure. LLINs made from traverse knitted structures also exhibited good hole enlargement resistance when comparing fabrics made of filaments of comparable linear density and fabric areal density. By comparison, tulle knitted structures are susceptible to tearing, laddering and unravelling.

Abrasion is a progressive wear mechanism that results in slippage, lateral deformation and eventually breakage of filaments within the knitted fabric structure. Although individual holes caused by abrasion are initially small in size [15], they have potential to enlarge, and abraded areas are obviously susceptible to damage by other mechanisms. Abrasion resistance depends on many factors including polymer type, filament linear density (denier), choice of knitted structure and finishing processes [26, 27]. In the present study, LLINs made of monofilament yarns were generally more abrasion resistant than those made of multifilament. This can be attributed to the greater propensity for slippage and breakage of the much finer individual filaments within multifilament yarns.

Seam strength is determined by fabric construction and areal density, the tensile properties of the yarn used to make the seam, stitches per unit length and the seam construction [28]. Clearly, there is a range of variables and a ‘one size fits all’ approach across the variety of LLIN types is likely to yield differences in the seam strength. Seams are responsible for holding the panels of a LLIN together, and breakage usually means the formation of a large hole.

Good practice for seams normally requires selection of a stitching yarn strength and density (stitches per inch) that conforms to the following equation for seam strength of lockstitch seams [29]:

1 $${\mathrm{F}}_{sL} =1.5 \times\upbeta \times\upgamma$$

Here β represents the stitches per inch, γ is the thread strength (lbs) and 1.5 is factor based on the loop strength ratio for most sewing threads. In addition:

- The sewing thread used in the seam should be of similar physical properties to that used to make the netting fabric in terms of breaking strength, ultraviolet light and mould resistance.
- The seams should be sewn at a tension that produces no loose or projecting loops or seam puckering.
- Double stitched seams should be employed, as they enable higher bursting strength than single stitched seams, and additional protection is provided if one row of stitches fails in the seam.

Therefore, in addition to the new suite of textile test methods, comprising: snag strength test, bursting strength, hole enlargement resistance and abrasion resistance, implementation of revised seam construction specifications are advisable to minimize the risk of catastrophic seam failure in the field.

## Conclusions

LLINs have to remain in good physical condition if they are to function properly as a vector control product. A critical step for ensuring LLINs are capable of maintaining physical integrity is to assess their ability to resist damage in the laboratory before use and to implement associated minimum specifications. A new suite of four textile testing methods specifically developed for this purpose has been developed and successfully cross validated by five laboratories. Additional guidance is also provided in relation to seam construction in order to prevent catastrophic failure during use. It is recommended to implemenent the full suite of textile testing methods as well as seam construction requirements as part of new specifications for LLINs. The WHO prequalification programme is looking at expanding testing to ensure specifications are fit for purpose. The suite of textile testing methods should be carried out on LLINs as a quality control measure at factory level prior mass distribution. Collectively, this would address a long-term omission in the assessment of existing LLIN vector control products, and with agreed minimum standards, provide a basis to improve quality and value, as well as encourage innovation of better performing products.

Current LLIN products are not the same in terms of their inherent ability to resist the primary mechanisms of damage encountered during normal household use. Various factors influence the ability of LLINs to resist mechanical damage such as the choice of polymer materials, yarn linear density (denier), mesh count and knitted fabric construction. This highlights the important role that textile science and technology will play in the design of higher performance LLIN products in the future.
